# Few-Shot Learning for Image-Based Nonintrusive Appliance Signal Recognition

**DOI:** 10.1155/2022/2142935

**Published:** 2022-08-23

**Authors:** L. Matindife, Y. Sun, Z. Wang

**Affiliations:** ^1^Department of Electrical and Electronic Engineering Science, University of Johannesburg, Auckland Park 2006, South Africa; ^2^Department of Electrical Engineering, University of South Africa, Florida 1710, South Africa

## Abstract

In this article, we present the recognition of nonintrusive disaggregated appliance signals through a reduced dataset computer vision deep learning approach. Deep learning data requirements are costly in terms of acquisition time, storage memory requirements, computation time, and dynamic memory usage. We develop our recognition strategy on Siamese and prototypical reduced data few-shot classification algorithms. Siamese networks address the 1-shot recognition well. Appliance activation periods vary considerably, and this can result in imbalance in the number of appliance-specific generated signal images. Prototypical networks address the problem of data imbalance in training. By first carrying out a similarity test on the entire dataset, we establish the quality of our data before input into the deep learning algorithms. The results give acceptable performance and show the promise of few-shot learning in recognizing appliances in the nonintrusive load-monitoring scheme for very limited data samples.

## 1. Introduction

The nonintrusive load monitoring (NILM) [1–4] has achieved high automatic recognition of appliances' operational status, through the measurement of the complex signal from a single point on the mains supplying the building. Today, a number of issues attribute to the successful implementation of NILM appliance recognition systems. These issues include higher data acquisition throughput and more storage hardware, better simulation software, better imbedded implementation hardware, and the use of deep learning (DL) algorithms. Machine-learning (ML) algorithms premised on hand-engineered features achieve acceptable performance when the data count is relatively low. DL algorithms inherently achieve better feature extraction, and usually, the data count is very high. The performance of DL algorithms greatly outperforms that of the rest of hand-engineered algorithms. As a way of increasing the data count, data augmentation methods [5, 6] normally complement data obtained from direct measurement. Data processing in MILM systems is either time series (TS) [2] or the image (IM) [7, 8] equivalent of the appliance TS signals. The IM data approach aims at availing more appliance features in a smaller space for improved but simpler identification through convolutional neural network (CNN) computer vision (CV). We can improve the feature base of the IM dataset to mimic a larger dataset by implementing multivariate IMs, information, and IM fusion inputs into the DL algorithms [9, 10]. However, the cost of acquiring large amounts of data becomes high, mainly in terms of increased data acquisition time and increased storage memory requirements. Computation time and dynamic memory usage become higher during model execution in such situations. In addition, if used, data augmentation and fusion inherently add to the complexity of the IM preprocessing stages.

Few-shot learning (FSL) [11, 12] allows the successful implementation of ML recognition algorithms on very limited input datasets. In FSL, ML algorithms mimic the ability of humans to identify an object in a different or new situation, based only on minimal or no prior interaction with that object [11]. The ability to learn-to-learn (also known as meta-learning (MEL)) from a previous situation makes it possible to achieve this type of recognition capability. Thrun & Pratt [13] give a detailed description of the learn-to-learn process and the expected outcomes of a machine or algorithm that can learn-to-learn. MEL is achieved by using two algorithm approaches, namely, metric [11, 14–16] and gradient-descent [17, 18] learning approaches.

In literature, usually to evaluate the effectiveness of FSL algorithms, we have a comparison to Baseline and Baseline++ models. The building of Baseline models is through a normal transfer learning classification approach. Baseline++ models are an improvement on the standard Baseline models [14, 16, 19]. The Bayesian, *k*-nearest neighbours (kNN), and the Siamese network [20, 21] are successful early one-shot learning attempts to classify IMs. The Bayesian method learns the relationship between inputs by using a probabilistic approach to relate the attributes of these inputs. In KNN (*K* = 1), the algorithm maps the feature space for two input IMs such that any new input IM outcome is determined by its nearest neighbour. The MEL approach can considerably enhance the performance of Bayesian, KNN, and Siamese classification networks.

There has been successful application of FSL in areas such as robotics, natural language processing, acoustic signal processing, drug recovery, and CV [12]. However, there is scant documentation of FSL as specifically applied to NILM classification [20–22]. The difficulty in realizing one-shot classification has slowed its adoption in NILM systems[23]. Nonetheless, we show some FSL literature developments in NILM. The authors in Ref. [22] proposed the classification of a number of appliance signals using FSL. In Ref. [22], the authors make a comparison of the few-shot performance of the KNN, decision trees (DT), random forest (RF), and long short-term memory (LSTM) models. The models gave *F*1-scores that varied from 0.898 to 0.930, which is an assessment of a model's accuracy on a given dataset. Moreover, the algorithms are not MEL and use power series (PS) appliance signal lengths determined by a sliding window to capture the minimum appliance activations. In Ref. [20], the authors proposed the Siamese neural network for classifying V/I trajectory images. Training of the Siamese networks is based on one-shot pairs of the same and different label V/I trajectory IMs. Similar appliances belonging to the training set form a cluster, with unrecognized appliances forming their own new cluster. The density-based clustering of appliances with noise (DBSCAN) technique provides improved clustering [20]. However, there is still need to improve on the classification performance of the system in [20], as some appliances are not recognized.

In this article, we propose the development of metric-based Siamese and prototypical FSL algorithms for the classification of the limited disaggregated appliance signal images in the NILM recognition. Contrary to the method in Ref. [22], we attack the FSL from a MEL CV perspective to improve the appliance signals' classification performance. We obtain a very limited in-house input dataset for the intended experiment from fourteen PS appliance operational status signals transformed into the signal IM equivalent form using Gramian angular summation fields (GASFs) [7, 8]. The fourteen appliances considered in this article are made up of four light-emitting-diode (LED) mains lamps, two compact fluorescent (CFL) mains lamps, three modes of HP laptop operation, a refrigerator, a microwave oven, a desktop computer, a two-plate cooking stove, and a kettle. The contributions of this article are as follows:

- The development of high NILM appliance classification Siamese and prototypical FSL algorithms based on CV. This results in a reduced dataset to as low as one appliance signal sample per class (one-shot) that effectively eliminates the negative voluminous data-related issues to NILM classification systems.
- To establish the level of closeness between data samples by carrying out a similarity test on the entire dataset. The similarity test value should be *S*_*TV*_ ≥ 0.6. A lower similarity value would require data preprocessing. We arrive at a value of 0.6 by attempting to have data that are easily separable at first sight, leaving the extra-involved 0.4 separation to a better-designed metric-learning network structure.

We organized the remaining parts of the paper as follows. In Section 2, we present the similarity, loss functions, and meta-learning theory.  Section 3 gives a presentation and detailed design of the proposed system. We also explain how the data are organized. In Section 4, we present and give a discussion of the experimental results.  Section 5 gives a closure to the article through the conclusion.

## 2. Similarity Theory, Loss Functions, and Meta-learning

### 2.1. Similarity Theory

Standard ML method classifies objects by assigning a probability or class value to the object in relation to the known class labels. The ML algorithms sample a large number of labeled objects to be able to achieve a good classification. In contrast, the ML similarity approach assesses the level of similarity between two objects to show whether they belong to the same class.

**Definition 1 .**

Two sets *X* and *Y* give a Cartesian product between them of *X* × *Y*={(*x*, *y*) : *x* ∈ *X* and *y* ∈ *Y*}. If *X*=*Y*, the Cartesian product of *X* with itself is *X* × *X*=*X*^2^. A similarity measure *S* [24] is a function with nonnegative real values defined on the Cartesian product *X* × *X*:

(1) $$\begin{array}{c} S:X\times X⟶ℛ, \end{array}$$

such that the following three properties are satisfied:

1. ∃*s*_0_ ∈ ℛ : −*∞* < *S*(*x*, *y*) ≤ *s*_0_ < +*∞*, ∀*x*, *y* ∈ *X*
2. *s*(*x*, *x*)=*s*_0_, ∀*x* ∈ *X*
3. *s*(*x*, *y*)=*s*(*y*, *x*), ∀*x*, *y* ∈ *X*

If in addition,

1. *s*(*x*, *y*)=*s*_0_↔*x*=*y*
2. *s*(*x*, *y*)*s*(*y*, *z*) ≤ [*s*(*x*, *y*)+*s*(*y*, *z*)]*s*(*x*, *z*)∀*x*, *y*, *z* ∈ *X*.

The *S* is called a metric similarity measure [24].

The aim of metric similarity measure learning is to decrease the separation between the embedding points of similar inputs. To evaluate the similarity between objects, various similarity measures exist [25]. These include the Euclidean distance, the Pearson's correlation for time series data, the Mahalanobis distance, which is a variation in the Euclidean distance with correlation, Dynamic Time Warping for time series comparison, cosine distance, Jaccard, and Tanimoto similarity measures [25]. For discrete systems, the similarity measures include the Jaccard index, Sorenesen coefficient, and the symmetric difference [24]. The most popular distance metric in ML is the Euclidean. The separation between the embedding of points of dissimilar inputs is to be increased. If {*x*_*i*_} ∈ ℝ^*n*^ is a number of points, then for similar points *x*_*i*_ and *x*_*j*_ to satisfy similarity [26],

(2) $$\begin{array}{c} S:\left(x_{i},\;x_{j}\right)\in S. \end{array}$$

The distance learning function in (2) is able to bring similar points together and dissimilar points apart in the embedding space:

(3) $$\begin{array}{c} d\left(x,y\right)=d_{A}\left(x,y\right)=|x-y_{A}|=\sqrt{{\left(x-y\right)}^{T}A\left(x-y\right)}, \end{array}$$

where *A* is an optimum matrix. For metric learning, *A* is semi-positive definite, *A* ≥ 0, and when *A* = *I*, we obtain the Euclidean distance [25, 26].

### 2.2. Loss Functions

The constructive loss function is well suited to metric learning. This function works on pairwise data samples and optimizes the training based on closeness or the absence of it between the samples. Let *d*_*W*_(*x*, *y*)=*D*_*W*_ be the parameterized Euclidean distance between the outputs embedding of *x*_*i*_,  *x*_*j*_ as defined in (3). Then, the contrastive loss function is

(4) $$\begin{array}{c} L_{\text{contrastive}}=\left(1-Y\right)\frac{1}{2}{\left(D_{W}\right)}^{2}+\left(Y\right)\frac{1}{2}\text{max}{\left(0,m-D_{W}\right)}^{2}, \end{array}$$

where *m* > 0 is a margin. The margin is the radius encapsulating the embedding area, such that dissimilar samples will only contribute to the loss function if the metric distance is within the margin. *Y* is a binary indicator for the samples. As an example for a pair of similar inputs, this value is 0 and, for two dissimilar inputs, it is 1 [27]. In other words, the first part of (3) deals with similar points, while the last part of the same equation deals with dissimilar points.

The triplet loss (TL) [28] is another widely used metric loss function mainly in Siamese learning. In this particular case, we identify three input images (an Anchor (AC), a Positive (PO), and a Negative (NE)) each passed through one of three CNN shared weights parallel models (Siamese network) with the three embedding models concatenated. The TL attempts to bring the embedding of the AC and PO closer, while it pushes further apart those of the AC and NE. The distance between the AC encoding (*f*(*AC*)) and the PO encoding (*f*(*PO*)) is

(5) $$\begin{array}{c} d\left(AC,PO\right)=\left\|f\left(AC\right)-f{\left(PO\right)}^{2}\right\|. \end{array}$$

The distance between the AC encoding (*f*(*AC*)) and the NE encoding (*f*(*NE*)) is

(6) $$\begin{array}{c} d\left(AC,NE\right)=f\left(AC\right)-f{\left(NE\right)}^{2}. \end{array}$$

The aim is to have *d*(*AC*, *PO*) ≤  *d*(*AC*, *NE*), that is,

(7) $$\begin{array}{c} d\left(AC,PO\right)-d\left(AC,NE\right)\leq \;0. \end{array}$$

To avoid a trivial solution for (6) in which case the embedding would be equal, it is necessary to incorporate a hyperparameter margin ∝ as shown in the following equation:

(8) $$\begin{array}{c} d\left(AC,PO\right)-d\left(AC,NE\right)+\;\propto \;\leq \;0. \end{array}$$

The margin makes sure that there is an appreciable separation between *d*(*AC*, *PO*) and *d*(*AC*, *NE*). The TL is

(9) $$\begin{array}{c} L\left(AC,PO,NE\right)=\text{max}\left(d\left(AC,PO\right)-d\left(AC,NE\right)+\;\propto ,\;0\right). \end{array}$$

### 2.3. Meta- and Few-Shot Learning

The meta-learning system works by training a large number of unrelated tasks. Each training task learns to classify images in a query set from the support set of that task (each task has its own support set and query set images. However, all tasks have the same classes and samples in the support set. The query set has the same number of samples across tasks. The images in each task are different). The test task that contains entries completely different from the training tasks would have learned a way of classifying the query test set from the support test set. The generation of a large number of training tasks (to optimize the training model) can only be achieved from datasets that have a large number of classes and relatively few samples per class. In few-shot learning, two popular datasets contain a very large class count to meet the requirements of training few-short models. The first is the Omniglot dataset comprising 50 alphabets with varying hand-written character (class) numbers each and having 20 samples per character for a total of 1623 characters (classes) [14, 29]. The class count in the Omniglot dataset is high, but the samples per class are few. On the other hand, the Modified National Institute of Standards (MNIST) dataset used as a baseline for testing image ML algorithms has only 10 classes but many samples per class. The second is the miniImagenet dataset that uses 100 image classes divided into 80 training and 20 testing samples [14]. Each class in the miniImagenet has 600 samples. The authors in [23] used the few-shot method on the full Imagenet dataset for 1000 classes to achieve high accuracy, having just a few samples per class that varied from 1 to 3. Although these datasets provide a baseline for developing and testing successful few-shot algorithms, in this article, we have produced a more applicable in-house NILM dataset. As in Ref. [23], only in respect to the number of samples per class, our in-house NILM dataset is processed in three ways: (1) 14 (Way) × 3 (Shot), (2) 14 (Way) ×2 (Shot), and (3) 14 (Way) × 1 (Shot). We evaluate and test the performance of our system on these three data presentations; however, our ultimate goal is the 1 (Shot) model since this allows for the minimum possible data sample without considering the zero-shot scenario.

With *N* (Way) classes in the support set and each class having *K* (Shot) images for a total of *N* × *K* support set images, we aim to classify an image out of *Q* images in the query set. The classification problem is one-shot, three-shot, or five-shot when the value of *K* is one, three, or five, respectively. In few-shot learning, the dataset samples (*K*) are usually less than ten samples. A special case arises when *K* is zero (zero-shot learning (ZSL)). ZSL first learns a projection of labeled (train) data into a new feature space. It then places projections of unseen (test) data into the same feature space and evaluates the distance or similarity value between the train and test entries to establish their relationship [30, 31]. Few-shot is an inductive transfer learning process where we optimize a new task based on previous knowledge about a different task with the same underlying structure. Metric-learning algorithms that include the MatchingNet [14], ProtoNet [15], and RelationNet [16] evaluate the distance or similarity function between images. By so doing, the algorithms can group images together that have smaller distance functions between them.

#### 2.3.1. Siamese Network

The Siamese network comprises single-input two-parallel-shared weight CNN networks that are both connected to the same distance function block that in-turn connects to a loss function block. The output of each CNN network before the distance function block is a vector space containing the features or embedding of each input. The similarity between the input embedding points is evaluated in the distance function block through the *L*2 norm (Euclidean distance) *| ***x** − **y**_2_*|*, *L*1 norm *| ***x** − **y**_1_*|* or cosine similarity cos(**x**, **y**). The loss function implements the contrastive or triplet loss-based model optimization during training. The Siamese network is most appropriate for one-shot learning [20, 21].

#### 2.3.2. Matching Network

The matching network uses two different functions *g*_*θ*_ and *f*_*θ*_ to extract the embedding of the support and query sets, respectively. The cosine similarity function compares each support set image features (embedding) to the query set features, followed by softmax classification. Full context embedding (FCE) through LSTM networks allows the production of an embedding that is the resultant of all the support set image features. FCE improves the performance of the MatchingNet especially in complicated situations [14]. In (9), we show the relationship between the query test sample $\hat{x},$ and query predicted label $\left(\hat{y}\right)$ from classification as [11, 14]

(10) $$\begin{array}{c} P\left(\hat{y}|\hat{x},S\right)=\sum_{i=1}^{k}a\left(\hat{x}|x_{i}\right)y_{i}, \end{array}$$

where *k* is number of support set samples, *x*_*i*_, *y*_*i*_ represent the support set object-label pairs, *S*={(*x*_*i*_, *y*_*i*_)}_*i*=1_^*k*^, and *a* is the attention mechanism. The attention mechanism *a*(., .) chooses the most significant attributes in evaluating the similarity in embedding points.

#### 2.3.3. Prototypical Networks

This is a less complex metric-learning algorithm that is capable of higher performance that matching networks. In this algorithm, we first find the prototype (*c*_*k*_) mean class of every object in that class. Secondly, we realize the softmax classification of the test object (query) by establishing the Euclidean distance between the query and prototype embedding [15].

The calculation of the prototype point is as follows:

(11) $$\begin{array}{c} c_{k}=\frac{1}{\left|S_{k}\right|}\sum_{\left(x_{i},y_{i}\right)\in S_{k}}f_{\emptyset }\left(x_{i}\right), \end{array}$$

where *S*_*k*_ represents the *k* class support point, *x*_*i*_ is feature point with label *y*_*i*_, and *f*_∅_ is the embedding function having ∅ trainable parameters. The evaluation of the query where *d* is the Euclidean distance between the query and prototype class is as follows [15]:

(12) $$\begin{array}{c} p_{\emptyset }\left(y=k|X\right)=\frac{\exp\left(-d\left(f_{\emptyset }\left(X\right),C_{k}\right)\right)}{\sum_{k}\exp\left(-d\left(f_{\emptyset }\left(X\right),C_{k}\right)\right)}. \end{array}$$

A bigger training class count than that for testing normally achieves better results, but maintains the class samples the same in both training and testing situations [11]. The training episode for the negative log-probability *𝒥*_∅_=−log*p*_∅_(*y*=*k|X*) through SGD where *k* is the true class [15] is given in Algorithm 1.

#### 2.3.4. Relation Network

In this model, there is concatenation of the support set (*f*_*φ*_(*x*_*i*_)) and query set (*f*_*φ*_(*x*_*j*_)) feature maps produced by the same embedding function. The function that concatenates the feature maps is ∁(*f*_*φ*_(*x*_*i*_), *f*_*φ*_(*x*_*j*_)). The concatenated result is processed in the relation module to output a similarity measure (relation score) between *x*_*i*_ and *x*_*j*_ of value 0 to 1. The number of relation scores depends on the number of classes in the support set. Equation (12) shows the expression for the relation score [16]:

(13) $$\begin{array}{c} r_{i,j}=g_{\emptyset }\left(∁\left(f_{\varphi }\left(x_{i}\right),f_{\varphi }\left(x_{j}\right)\right)\right),i=1,2,\ldots ,∁, \end{array}$$

where ∁ is number of classes in support set, *x*_*i*_ is support set objects, and *x*_*j*_ is query set entry during model training.

#### 2.3.5. Model-Agnostic Meta-Learning (MAML)

The MAML [18, 32] is unique among meta-learning methods since it is implementable on any gradient-descent model. To address the meta-learning problem, if the MAML model successfully solves a previous task, then it should learn to deal with a new task in a faster way with improved performance. The MAML seeks to have a learnable parameter *θ* move close to the optimized *θ*_*i*_^*∗*^ parameter values of different tasks [18]. This *θ* becomes the initialization value, which is specific task fine-tuned. The *θ* trajectory involves the continuous optimization of the loss functions *L*_*i*_ for the tasks [18]. We define a task as T_*i*_={*p*_*i*_(*x*), *p*_*i*_(*y|x*), *L*_*i*_} that shows the distribution over the input *p*_*i*_(*x*), the distribution over the labels given the input *p*_*i*_(*y|x*), and the loss *L*_*i*_. The distribution of tasks is *p*(*T*). If *f*_*θ*_ represents the classification model, then the training set loss is

(14) $$\begin{array}{c} \underset{\theta }{\min}\sum_{T_{i}∼p\left(T\right)}L_{T_{i}}\left(f_{\theta }\right). \end{array}$$

The gradient descent optimizes the loss as

(15) $$\begin{array}{c} \underset{\theta }{\min}\sum_{T_{i}∼p\left(T\right)}\nabla_{\theta }L_{T_{i}}\left(f_{\theta }\right). \end{array}$$

When the learning rate is *α*, the complete gradient-descent update is

(16) $$\begin{array}{c} \theta_{i}^{\prime }=\theta -\alpha \nabla_{\theta }L_{T_{i}}\left(f_{\theta }\right). \end{array}$$

Training of the model to minimize *L*_*T*_*i*__(*f*_*θ*_*i*_′_) then follows in the meta-objective as

(17) $$\begin{array}{c} \underset{\theta }{\min}\sum_{T_{i}∼p\left(T\right)}\nabla_{\theta }L_{T_{i}}\left(f_{\theta_{i}^{\prime }}\right)=\sum L_{T_{i}}\left(f_{\theta }-\alpha \nabla_{\theta }L_{T_{i}}\left(f_{\theta }\right)\right). \end{array}$$

Covering all the tasks, the stochastic gradient descent (SGD) updates the meta-optimization parameter *θ* as

(18) $$\begin{array}{c} \theta \leftarrow \theta \beta \nabla_{\theta }\sum_{T_{i}∼p\left(T\right)}L_{T_{i}}f\left(\theta_{i}\right), \end{array}$$

where *β* is the meta step size [18–33].

## 3. Methodology

### 3.1. Proposed System

Based on literature review, compared to other metric networks, the matching network is more involved to realize [14] and normally achieves less performance. Due to this, we do not consider the matching network for application in this article. Due to its simplicity, it is possible to implement a relational metric-learning network. However, for now we only explore the prototypical network.

Appliance activation periods vary considerably, and this can result in imbalance in the number of appliance-specific generated signal images. There is no effect on the performance of few-shot prototypical metric-learning networks by this data imbalance. In this article, we propose the application of prototypical networks. Prototypical networks only produce a prototype (average) value embedding point of the samples in each class during training. A comparison is made of the average prototypes with a test embedding point through the Euclidean distance metric. We first carry out a similarity test on the entire dataset to establish the level of similarity between the data samples. The application stage of the prototypical network will require a similarity test value of at least 0.6 to increase the accuracy of our few-shot learning model.

**Figure 1:**
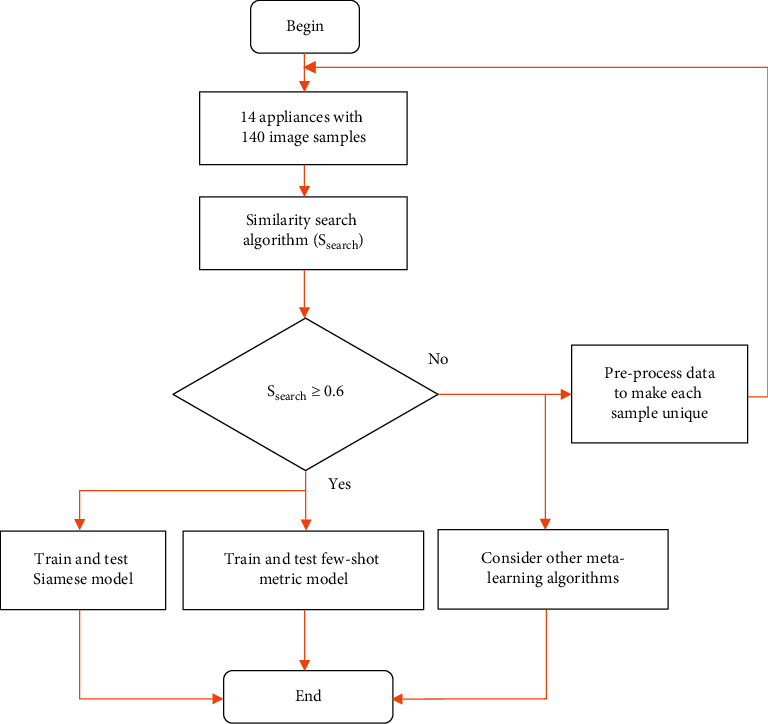
Proposed system with data presimilarity test.

We give the flowchart of the proposed system in Figure 1. Our proposed system allows for quick determination of the suitability of disaggregated appliance data for metric learning before the actual few-shot learning. By so doing, we are able to preprocess the data before conversion into acceptable TensorFlow file formats, which can result in improved model training. We assign an acceptable data similarity value in the overall data similarity search.

**Figure 2:**
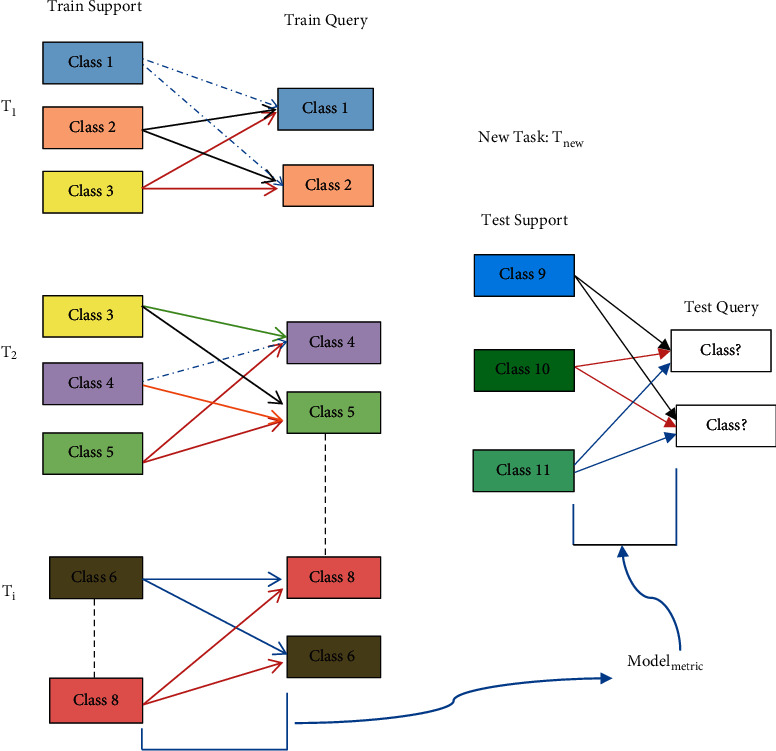
Few-shot metric-learning nonintrusive appliance recognition framework.

In the proposed train and test few-shot metric model block exploded in Figure 2, we seek to address the recognition of limited appliances signals by employing a model (Model_metric_) based on testing the similarity or dissimilarity between a known appliance signal image in the support set (*D*_support_) and an unknown disaggregated appliance signal image in the query set (*D*_query_). A conventional image-based deep learning neural model would require training by a very large sample count in (*D*_support_). The proposed system includes a training dataset (*D*_train_) to train the Model_metric_. Training of the model is through a larger base set split into a specific number of different tasks ({**T**_**i**_, *i* ∈ *h*}for1 ≤ *i* ≤ *h*), for *h* tasks to optimize the loss function. The Model_metric_ is the prototypical network. A 1 shot Siamese model can also be realized. The training allows for the realization of a model that learns to learn to place the embedding of similar classes together in the test task.

### 3.2. Dataset Preparation

**Figure 3:**
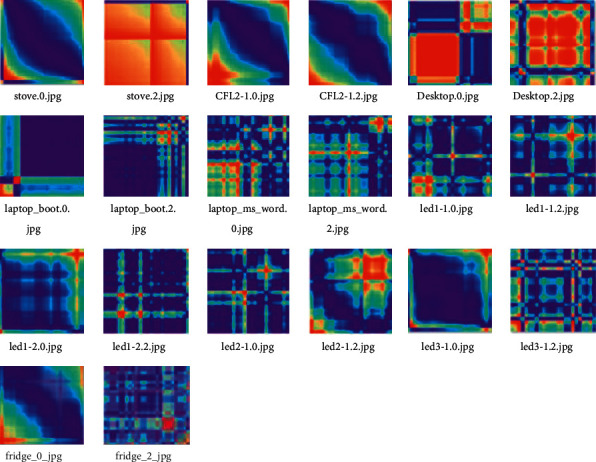
Two samples each of the ten (10) train support classes.

The dataset is made up of fourteen appliance categories or classes placed in an ALL_IMAGES main directory on the computer. These appliances include four light-emitting-diode (LED) mains lamps (LED1-1 (5W), LED1-2(5W), LED2-1 (5W), and LED3-1 (5.5W)), two compact fluorescent (CFL) mains lamps (CFL1-1(12W), CFL2-1(14W)), three modes of HP laptop operation (laptop_boot, laptop_ms_word, laptop_video), a refrigerator (fridge), a microwave oven (microwave), a desktop computer (desktop), a two-plate cooking stove (stove), and a kettle (*K*). A sample of our raw few-shot train support dataset is shown in Figure 3 and is comprised of GASF IMs initially in RGB format and shape 400  × 400 × 3. In Figure 3, we have shown only two samples out of ten samples per class. Using a PA1000 Tektronix [34] power analyzer in a laboratory setup, we measure the operational TS signals over the complete activation of the appliance. We then transform the appliance's activation signals to IM equivalent by using GASF.

**Figure 4:**
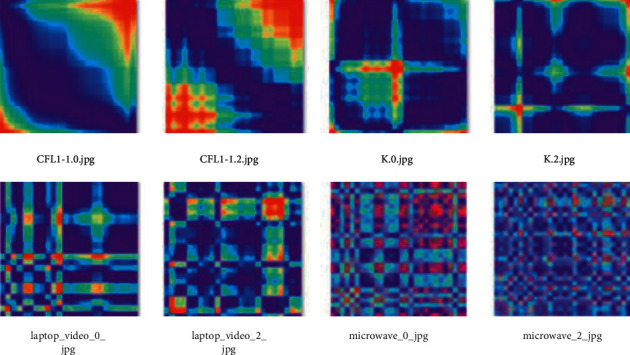
Two samples each of four (4) test support classes.

Figure 4 shows the images used in the test support and never seen before by the few-shot model.

**Figure 5:**
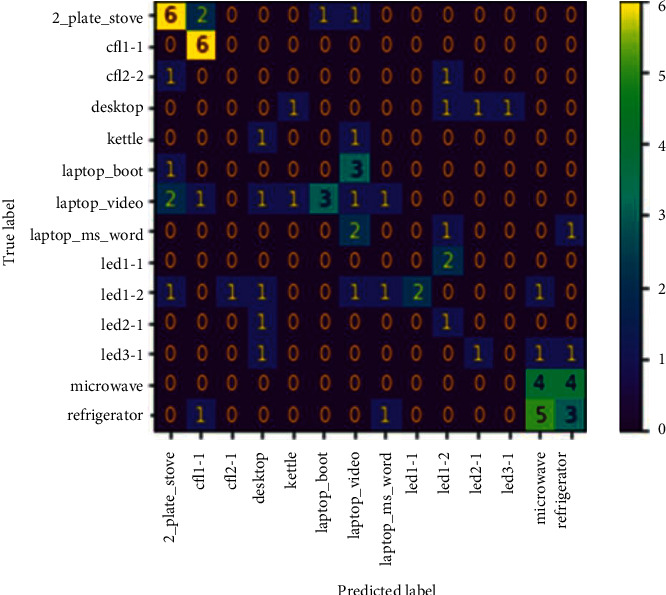
Similarity test confusion matrix.

As clearly seen in Figures 3 and 4, the sample images have different features and this property is used to successfully train and test the few-shot meta-learning model. It is important at this point to note that for the similarity test model, the samples in Figures 4 and 5 are considered one dataset, which is then split using the sklearn train_test_split. Converting the RGB images to grayscale and reducing image size helps to decrease the complexity of developed algorithms, speed up the process, and use less computation resources.

The use of both the omniglot and miniImagenet datasets for evaluating developed FSL algorithms is widespread. We observe that typical file formats in FSL algorithms include the NumPy array (.npy), tar.gz (.tgz), or just straight image file folder. However, the IMs in these FSL algorithms are normally converted to grayscale (*L*) and resized to 28 × 28. The .npy grayscale images have an initial shape of 28 × 28. To take advantage of existing few-shot coding in literature, we prepare our custom dataset more or less in the same manner as for the omniglot and niniImagenet datasets. TFRecords files present data in binary record sequences. TFRecords is the recommended TensorFlow data format. We do not evaluate our final models on TFRecords as the conversion of various data formats to TFRecords requires different coding approaches. However, we do experiment with TFRecord files.

For each appliance, we are able to capture at least ten activation signals, which results in ten signal IM samples per appliance class. The total number of resized IMs per one measurement exercise is 140. When we take the NumPy route, the produced IM NumPy array is reshaped to [number of classes, samples per class, IM width, IM height, channels] to give shape (14, 10, 28, 28, 1). We then split this into train, test, and validation data. We follow the directory structure of the omniglot dataset to achieve the NumPy and reshape above. The execution of the create-miniimagenet.py converts the image miniimagenet folder into train.npy and test.npy. On the other hand. the helper.py script converts the omniglot dataset to .npy.

We then run a custom-developed script to convert our ALL_IMAGE folder into train.py and test.py. In some instances, we performed the data split by producing train, test, and validation CSV files that contain the IM file name and label. The labeled IMs in this case are stored in their own separate folder.

### 3.3. Training Procedure

Due to the extensive coding required for the FSL algorithms, we had to experiment with code examples from numerous GitHub repositories and from keras.io code examples [35, 36]. We implemented the code in both python and keras in IPython and Jupyter platforms, respectively. In training some code, we used the Google Colaboratory (Google Colab.) notebooks platform in which we could easily install such packages as PyTorch. Google Colab also allowed us to use the graphics processing unit (GPU) facility not available on our HP 650 Notebook to speed up training. However, in these codes, we modified the utilities (utils) data handling part to accommodate our in-house datasets. We also modified or added code for specific data results' visualization and experimented with various hyper-parameters. In some instances, we experimented with different number of convolutional layers. We also experimented with different epochs, episodes, and different number of support and query set classes and shots. However, our target system was the 1-shot model.

We trained the similarity test model in colab, with data loaded into My Drive in Google Drive. The RGB images of size 400 × 400 are resized to 28 × 28. Training is performed with a train-to-test split ratio 0.75 : 0.25 and batch size of one. The train shape is (105 × 28 × 28 × 3), and test shape is (35 × 28 × 28 × 3). High numbers of batch size did not improve system performance, probably due to our limited training samples. The embedding model is a three-layer VGG 2D convolutional network with filter sizes 32, 64, and 128 from the input and kernel size of 3. During training, we experimented with various hyper-parameters and we would get slightly different classification results. However, for best classification results, we settled for the Adam optimizer with a learning rate of 1*e* − 3 as in [35], and we used the sparse-categorical-cross-entropy loss function.

To train the Siamese network, we create two directories each with four grayscale images of dimension 400 × 400 × 1. The directories are the 2_plate_stove and CFL1-1(12W) appliance classes. After preprocessing, the new data shape is 16, 2, 1, 200, 200, where 32 is the total sample size, 16 for 2 pairs input into the Siamese network. The 1, 200, 200 represents the new dimensions of the images in pgm format. As in Ref. [37], the base model consists of two 2D convolutional layers followed by flatten operation and two dense layers. The first 2D CNN layer had 6 filters each of size 3, ReLU activation, followed by max. Pooling (2, 2) and dropout 0.25. The second 2D CNN layer had 12 filters each of size 3, ReLU activation, followed by max. Pooling (2, 2) and dropout 0.25. The first dense layer is 128 units with dropout 0.1, and the last layer has 50 units with reLu activation. We used the contrastive loss and RMS optimizer. In addition, we developed a Siamese model based on the triplet loss function with a margin (alpha) of 0.2, 98 train grayscale samples and 42 grayscale test samples. We experimented with different image formats that proved to be difficult to implement in the coding of [37].

In the prototypical recognition system, the data were based on the RGB IMs of size 400 × 400. These IMs from a total IM count of 140 are reshaped to 28 × 28  × 3. Two approaches were then used to format the input data into the prototypical network. The first approach involved the internal model augmentation through rotation at different angles to obtain a final train set shape of (400, 28, 28, 3) and a test shape of (160, 28, 28, 3). The second approach took the 140 IM samples and reshaped to train set shape (10, 10, 28, 28, 3) and test set shape (4, 10, 28, 28, 3), where 4 represents the test classes and 10 the number of samples per class for the test set. The second approach implementable on CPU because of the low memory requirements provided the results captured in this article. It was necessary to combine the different modules available in GitHub to come up with a TensorFlow prototypical network [38], which was also executed in colab under GPU. The codes are executed with the SGD optimizer and a learning rate of 0.1. The recognition efficiency generally increases as the number of episodes increases. In the first approach, the highest accuracy was at 30000 episodes for a frame size of 1000. However, in the second CPU approach, the maximum episode count was 600 episodes.

## 4. Experimental Results

### 4.1. Similarity Search

In Figure 5, we see the appliance recognition results based on similarity.

To evaluate the suitability of our dataset for metric learning, we use the code in [35]. In this similarity test algorithm, we infer images of the same class as being similar, while those between classes are not. A requirement in model training is the pairing of images in the same class for the similarity test. One image is the AC and the other the PO [35]. There are 38 test samples out of the 140 total appliance IM samples. By pairing the test samples, we obtain 76 samples. For 14 classes, that translates to six (6) samples in the test set assigned to each class.

The 2_plate_stove and cfl1-1 attained one hundred per cent recognition. The refrigerator and microwave oven also attained high levels of recognition. However, their relative high powers especially with the inclusion of the refrigerator switching spike some activations are almost similar between the two. The system also had difficulty in recognizing between the different operating modes of the laptop. However, the system was able to cluster all sample points as laptop among laptop_boot, laptop_video and laptop_ms_word, which in itself represents the success of the similarity test. With improved network and data, the similarity test system has potential for attaining high classification and hence passes the test criteria of 0.6. We give the similarity loss plot in Section 4.4

### 4.2. Siamese Network 1 Shot Learning

In Siamese network training, we have two authentic (similar) images to which we assign an authentic label of 1 to the pair. For a pair of images between classes, we assign a not-authentic label of zero (0). During training, the input into the Siamese network is either the pair of authentic images or a pair of not-authentic images. The trained model provides the set coordinate of the embedding of each similar pair per class. In our case, we obtain the Siamese 1 shot experimental results on both the contrastive and triplet loss functions. When the contrastive loss function trained Siamese network is tested against the compact fluorescent lamp (CFL2-1(14W)), it returns a true target value of 1 as given in the part sample code:

  In [34]: x_testsia[target_index:target_index + 1, 0].shape
  Out [32]:
  (1, 1, 200, 200)
  In [34]: predsia = model.predict([x_testsia[target_index:target_index + 1, 0], x_testsia[target_index:target_index + 1, 1]])
  predsia = predsia <0.5
  print(“y_test[target_index]:”, y_testsia[target_index, 0] = = True, “pred:” ,predsia)
  y_testsia[target_index]: True predsia: [[True]].

In triplet loss Siamese model, the train set shape is (98, 28, 28, 1) and the test shape is (42, 28, 28, 1). On the other hand, a part test code for the triplet loss is

  In [15]: btch_size = 9
  epchs = 200
  steps_per_epch = int(x_train.shape[0]/btch_size)
  net.compile (loss = triplet_loss, optimizer“='adam”)
  _ = net.fit(
   data_generator(btch_size),
   steps_per_epoch = steps_per_epch,
   epochs = epchs, verbose = False,
   callbacks = [
    PCAEmdPlotter(
     plt, embedding_model,
     x_test[:40], y_test[:40])]).

**Figure 6:**
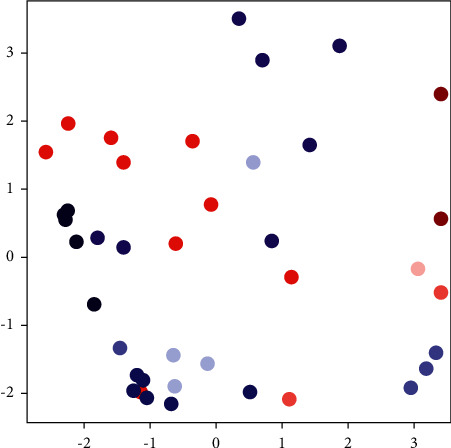
Triplet loss test embedding results.

We give both the contrastive and triplet loss plots in Section 4.4.  Figure 6 shows the results of the test imbedding in the triplet loss model.

From Figure 6, there is a tendency for clustering of the embedding in any class. These results here show that there is a need to increase the train dataset or redesign the model for deeper DL. These results are in synchronization with the results shown in Figure 5 where the model tries with effort to obtain the classification of different appliance signals that are almost the same.

### 4.3. Prototypical Network

The prototypical model achieves high accuracy early in the training and converges well. The train loss and accuracy plots are given in Section 4.4. To test the model, we specify different values of number of samples or shots in the support set (Ns), the different number of classes in the support set or ways (Nc), and the number of samples in the query set (Nq) whose class value is unknown.  Table 1 shows the relation between test support and query set sample entries per given appliance class number.

Table 1 gives all the few-shot learning test results collected for the prototypical network. In Tables 2, we show the summary of 2-way *k*-shot accuracy results for the prototypical network. In Table 2, the 2-way 7 shot gives the highest performance at 97.83% average test accuracy. Our data have four (4) test classes and ten (10) training classes. Hence, in the test and support class, the number of samples is either 4-way *k*-shot, 3-way k-shot, 2-way *k*-shot, or 1-way *k*-shot. Likewise, the training test set can vary from 10-way *k*-shot to 1-*k*-shot. The 1-way *k*-shot is a theoretical postulation, since in reality, we cannot have a model that trains to detect one class in our case. However, a multiple sample within one class is feasible. The limited classes in our experiment will result in slight model overfit of 100% train accuracy to 97.83%. The 2-way 1-shot system gives a reasonable average test accuracy of 91.343%. The results in Tables 1 and 2 are based on a 5-way k-shot training set. A 10-way *k*-shot training set did not produce satisfactory results. Comparing the performance of the prototypical network with the metric similarity search at the beginning, we see an agreement between the two systems. Table 2 also shows the average accuracies of the 3-way *k*-shot and 4-way k-shot FSL test models.

In Tables 2, we see that the test accuracy goes up as the k-shot value goes up in the support set. The number of classes is the same in the test support and query sets. The four test cases belong to the refrigerator, kettle, LED2-1(5W), and CFL2-1(14W) mains lamps.

We now make a comparison of the results of this article to published results that use the same datasets. In Table 3, we show training dataset (TRDS), training classes (TRCL), training query set (TRQS), training accuracy (TRACC), test dataset (TEDS), testing classes (TECL), and the testing accuracy (TEACC).

From Table 3, we can clearly see that with a very limited training dataset, our model achieves a higher training accuracy and higher test accuracy than from publications that use the same data in different model architectures. Reference [39] is IM classification based on the capsule network, while Reference [40] is classification based on the ConvNet VGG image classifier. From the similarity metric test results at the beginning, we see that we have a number of classes whose embedding is very close to each other. We need to investigate how we can improve further the accuracy of our model by considering such issues as hybrid MEL systems [41, 42]. There is need to consider the visualization of the embedding including the actual class objects and labels from the query set.

### 4.4. Model Loss Plots

**Figure 7:**
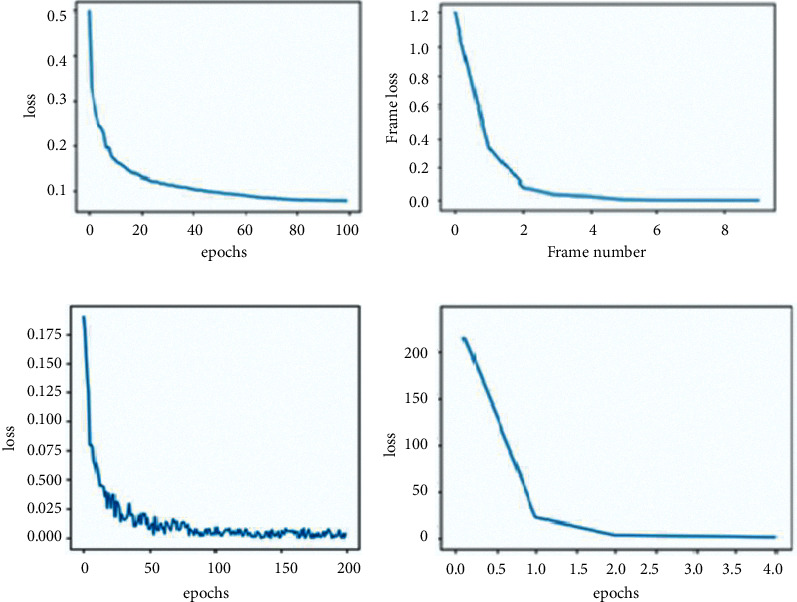
(a). Similarity network loss, (b) prototypical network loss, (c) triplet loss of Siamese network, and (d) constructive loss of the Siamese network.

Figure 7 gives the training loss plots for the models developed in this article. The plots show the attempt by the models to reach convergence trough stable training. Due to the limited training data points, the models tend to overfit; however, they do produce acceptable performance.

## 5. Conclusion

In this article, we investigate the application of few-shot learning in the form of a Siamese and prototypical network for the classification of disaggregated appliance signal images. By first carrying out a similarity test on the entire image dataset, we see that there are some appliances whose embedding is extremely close to each other. We observe a clear separation of embedding in other instances. We infer this information from the given confusion matrix. Nonetheless, the results show that we can achieve acceptable recognition of appliance signals using the Siamese and prototypical few-shot learning network. Two major challenges have been encountered in this study. The first is the inadequate number of available training classes so that the models could provide improved generalization. The second challenge is the closeness of some of the appliance signals to each other resulting in impaired discrimination between appliances. Program execution on normal CPU was extremely slow, or the system would just crash. Fortunately, we were able to make use of the GPU facility on Google Colab platform.

In future, we investigate the application of the MAML algorithm and the possible application of hybrid metric and gradient-descent few-shot learning methods for improved recognition performance. We will also consider increasing the training data classes and examples. Of particular interest is the improvement of the *N*-way 1-shot recognition setup.

## Figures and Tables

**Algorithm 1:**
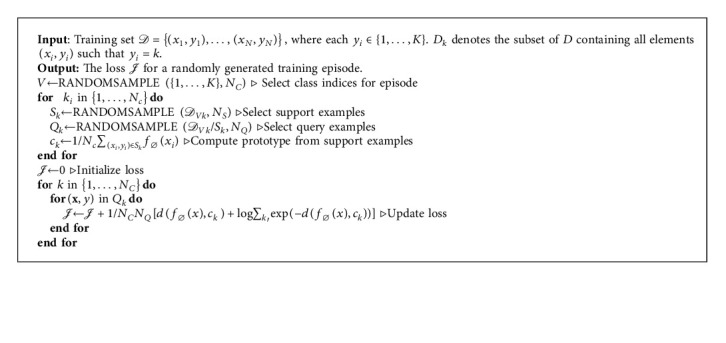
[15] Prototypical single training episode loss computation. *N* gives the number of training set samples, *K* gives the number of classes in the training set, NC ≤ *K* is the number of classes per episode, NS is the number of support examples per class, and NQ is the number of query examples per class. RANDOMSAMPLE(*S*; *N*) denotes a set of *N* elements chosen uniformly at random from set *S*, without replacement [15].

**Table 1:** Prototypical network few-shot learning test results.

Test	Ns	Nc	Nq	Test episodes	Avg. test accuracy %
1	1	2	9	600	91.343
				250	89.844
				100	91.167

2	2	2	8	600	94.406
				250	94.95
				100	94.50

3	3	2	7	600	94.976
				250	94.743
				100	95.429

4	5	2	5	600	96.10
				250	96.8
				100	94.7

5	7	2	3	600	96.722
				250	96.867
				100	97.83

6	8	2	2	600	97.417
				250	96.5
				100	96.5

**Table 2:** N-way *k*-shot accuracies for the prototypical network.

*N*-way	*k*-shot	Average accuracy (%)
2-way	1-shot	91.343
2-way	2-shot	94.95
2-way	3-shot	95.429.
2-way	5-shot	96.8
2-way	7-shot	97.83
2-way	8-shot	97.417
3-way	1-shot	90.543
3-way	2-shot	93.361
3-way	3-shot	93.643
3-way	5-shot	94.378
3-way	7-shot	95.333
3-way	8-shot	95.889
4-way	1-shot	88.17
4-way	2-shot	93.005
4-way	3-shot	93.374
4-way	5-shot	93.892
4-way	7-shot	93.708
4-way	8-shot	94.958

**Table 3:** Comparison with published results.

Method	TRDS	TRCL	TRQS	TRACC %	TEDS	TECL	TEACC %
							Avg:
Ours: Protonet	4	5	6	100.00	7	2	97.83
	4	5	6	100.00	8	2	97.41
	4	5	6	100.00	1	2	91.343

Reference [39]	22	5	—	75.00	8	2	93.75
Reference [40]	30	2	—	83.33	6	2	86.11

## Data Availability

The data used in this study are derived from public domain resources.
